# MicroRNA-29c-3p participates in insulin function to modulate polycystic ovary syndrome via targeting Forkhead box O 3

**DOI:** 10.1080/21655979.2022.2033014

**Published:** 2022-02-10

**Authors:** HongXia Chen, YunFeng Fu, ZiXiang Guo, XiaoDong Zhou

**Affiliations:** a Department of Gynecology and Obstetrics, The First Affiliated Hospital of Nanchang University, Nanchang, China; b Department of Gastroenterology, The First Affiliated Hospital of Nanchang University, Nanchang, China

**Keywords:** Polycystic Ovary Syndrome, insulin function, microRNA-29c-3p, Forkhead box O 3

## Abstract

MicroRNAs (miRNAs) are gene expression regulators and changes in miRNA levels are associated with diabetes, insulin resistance, and inflammation, the latter two of which are characteristic of polycystic ovary syndrome (PCOS). The purpose of this study was to explore the specific mechanism in which miR-29 c-3p participated in insulin function to regulate PCOS by targeting Forkhead box O 3 (Foxo3). Peripheral blood from PCOS patients and healthy volunteers were first collected, and the expression levels of miR-29 c-3p and Foxo3 were detected by reverse transcription quantitative polymerase chain reaction or Western blot. Then human granular tumor cell line (KGN) was treated with insulin, and transfected with plasmid vectors interfering with miR-29 c-3p or Foxo3 expression. Cell proliferation was detected by Cell counting kit-8 and plate cloning, and cell apoptosis was tested by flow cytometry. In addition, PCOS rat model was established. PCOS rats were injected with plasmids vectors interfering with miR-29 c-3p or Foxo3 expression, respectively. Pathological changes in ovarian tissues of rats in each group were observed by hematoxylin-eosin staining, and serum sex hormones and glucose metabolism-related indicators were detected. Finally, via bioinformatics website, luciferase digestion report assay was detected the targeting relationship between miR-29 c-3p and Foxo3. The experimental results showed that miR-29 c-3p was down-regulated in PCOS, but Foxo3 was up-regulated. Up-regulated miR-29 c-3p or down-regulated Foxo3 promoted KGN cell proliferation, inhibited apoptosis *in vitro*, restored PCOS rat sex hormone levels and improved glucose metabolism *in vivo*. These results suggest that miR-29 c-3p is involved in insulin function to improve PCOS by targeting Foxo3.

## Graphical abstract

**Figure:**
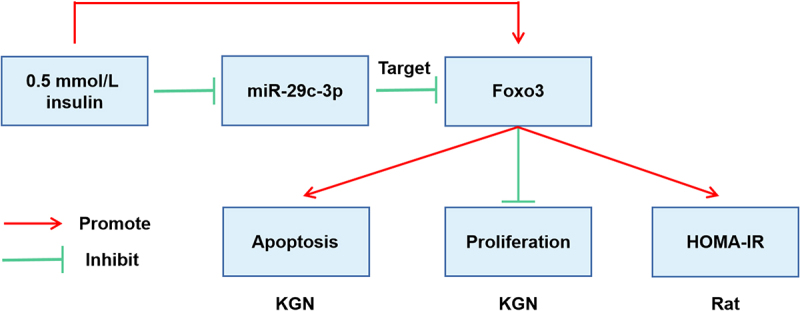

## 1. Introduction

Polycystic Ovary Syndrome (PCOS) is regarded as the most familiar endocrine and metabolic disorder in line with the standard of National Institutes of Health [1]. Around 5% women of childbearing age are diagnosed as PCOS [2]. Ultrasonic examination clarifies that the features of disease consist of insulin resistance (IR)/hyperinsulinemia, polycystic ovary, chronic anovulation, and hyperandrogenism [3]. Ovaries of PCOS patients comprise numerous small cysts, whose feature consist of elevated deposition of small follicles with a diameter of 4–7 mm and hypertrophy of the inner follicles [4], which is identified as the dominating source of anovulatory infertility. Moreover, PCOS frequently affects numerous organ systems and gives rise to diverse health complications, like acne, hirsutism, cardiac metabolic risk and menstrual dysfunction [5]. Interestingly, around 60%–70% PCOS patients emerge IR, which may be attributed to age, weight, or race [6]. In some fields of animal reproduction and human reproduction medicine, INS, and INS-mediated glucose uptake are rather vital for ovarian cells [7]. The pertinence of IR with PCOS remains to be roundly comprehended, since conventional INS pathway has no defects, involving INS binding and receptor expression [8]. Especially women with PCOS manifest dyslipidemia, remarkable IR, ascending risk of type 2 diabetes and restrained glucose tolerance [9]. As a consequence, these above discoveries insinuate that a latent pertinence of IR with PCOS, which requires for further elucidation [10].

MicroRNA (miRNA) is a small non-coding RNA, which usually modulates numerous target genes at the post-transcriptional level, thus taking a crucial effect in physiological and pathological processes [11,12]. Numerous studies have emphasized on the pivotal impact of miRNA on numerous biological processes, like cell advancement and metastasis of malignant tumors. Besides malignant tumors, multiple processes concerning in the pathogenesis of PCOS (advancement, differentiation, etc.) also emerge miscellaneous specific miRNA. A study puts forward that the pathogenesis of PCOS is positively associated with the expression profile of miRNA in rat ovaries, and the distinctions in 25 miRNAs of normal and polycystic ovary rats are rather prominent [13]. Meanwhile, other studies propose that miR-29 c-3p is silenced in diabetes mellitus [14,15]. Nevertheless, study of miR-29 c-3p in PCOS has almost remained undiscovered.

The Forkhead box O (Foxo) family is vital in the downstream of insulin signaling pathway [16], and have an active participation in miscellaneous cellular and physiological processes [17,18]. The subcellular localization and function of FOXO are under the control via post-transcriptional modifications, like acetylation and phosphorylation [19], and insulin is rather momentous in this process [20]. FOXO attenuates cell progression via inducing the transcription of pro-apoptotic genes, thus staying actively implicated in the process [21]. As a matter of reality, it has been proposed that Foxo3 is augmented in PCOS and accelerates progression of ovarian granulosa cells [22]. Nevertheless, the targeting connection of miR-29 c-3p with Foxo3 has not been studied.

In this study, the role of miR-29 c-3p was evaluated in the developmental context of PCOS. The expression of miR-29 c-3p in peripheral blood of PCOS patients and insulin-treated cells was observed. In addition, in the study, it was attempted to reveal the effects of miR-29 c-3p/Foxo3 on insulin-treated granular cells.

## 2. Methods

### 2.1. Participants and sample collection

This study has obtained authorization from Scientific Ethics Committee of The First Affiliated Hospital of Nanchang University. From October 2017 to December 2018, all participants in this study were women who had received Intracytoplasmic Sperm Injection (ICSI) or *In vitro* Fertilization (IVF) in The First Affiliated Hospital of Nanchang University. After obtaining of written informed consent, total 100 participants (50 patients with PCOS and 50 patients in the control group) were incorporated into this study. The diagnosis of PCOS patients was based on the standard of revised Rotterdam European Society for Human Reproduction and Embryology and American Society for Reproductive Medicine (2004). Patients with PCOS met at least two of following criteria involving polycystic ovary, androgen excess and chronic low ovulation or anovulation. In the control, patients conducting IVF or ICSI on account of male or infertile fallopian manifested as normal ovarian ultrasonogram, normal ovulation and regular menstrual cycle. All patients had no drug history influencing glucose and lipid metabolism, and no known aging diseases like endometriosis, androgen secreting tumor, Cushing syndrome and congenital adrenal hyperplasia. Homeostasis Model Assessment-Insulin Resistance (HOMA-IR) is an extensively utilized clinical index for evaluation of IR, whose definition is (FPG (mmol/L) × FIN (μIU/mL))/22.5. The critical point of IR was 2.14. The collection of peripheral venous blood of all subjects was for subsequent experiments.

### 2.2. Cell culture and insulin treatment

The culture of human granular tumor cell line (KGN) (Riken Biological Resources Center, in Ibaraki, Japan) was in Dulbecco’s Modified Eagle Medium/F12 (BD Biosciences, San Jose, CA) comprising 10% fetal bovine serum and 1% antibiotics. KGN cells were treated with 0.5 mmol/L insulin (Sigma-Aldrich) for 24 h, and then transfected.

### 2.3. The transfection of cells

On the grounds of the manufacturer’s instructions, the transfection of mimic/inhibitor NC, miR-29 c-3p mimic/inhibitor, sh-NC/Foxo3, miR-29 c-3p mimic + oe-NC/Foxo3 into KGN cells (all Shanghai Sangon Biotechnology Co. Ltd., Shanghai, China) was via Lipofectamine 2000 reagent (Invitrogen, Carlsbad, CA, USA).

### 2.4. Reverse transcription quantitative polymerase chain reaction (RT-qPCR)

The extraction of total RNA was from tissues and cells via TRIzol reagent (Invitrogen), and its reverse transcription into complementary DNA was via PrimeScript RT reagent kit with gDNA Eraser (Takara) on the grounds of the manufacturer’s instructions. Then, RT-qPCR was conducted on Light Cycler 480 system via SYBR Premix Ex Taq (Takara). The standardization of miR-29 c-3p and Foxo3 was separately via U6 and glyceraldehyde-3-phosphate dehydrogenase (GAPDH). The Analysis of data was via 2^−ΔΔCt^ method. Demonstration of primers is in Table 1.

**Table 1.** RT-qPCR primers

Genes	Primer sequence (5’–3’)
MiR-29 c-3p	F: GCG CGC GTA GCA CCA TTT GAAAT
	R: ATC CAG TGC AGG GTC CGA GG
U6	F: CTCGCTTCGGCAGCACA
	R: AACGCTTCACGAATTTGCGT
Foxo3	F: GTGGGGAACTTCACTGGTGCTAAG
	R: GGGTTGATGATCCACCAAGAGCTCTT
GAPDH	F: CGGAGTCAACGGATTTGGTCGTAT
	R: AGCCTTCTCCATGGTGGTGAAGAC

### 2.5. Western blot

The extraction of total proteins of tissues and cells was via Radioimmunoprecipitation Assay buffer solution (R0010, Solarbio, Science & Technology Co, Ltd., Beijing, China), with separation via sulfate polyacrylamide gel electrophoresis, electroblot onto polyvinylidene fluoride membrane (Millipore, USA), and seal with 5% skim milk. And then the incubation of the membrane with primary antibodies, and peroxidase-coupled secondary antibodies was exerted. The detection and analysis of immune response bands were via BIO-RAD ChemiDoc MP Imaging System and Image Lab Software. The dominating antibodies in Western blot were Foxo3 (1:1000) and GAPDH (1:1000) (all Abcam Inc., Cambridge, MA, UK).

### 2.6. Cell counting kit (CCK)-8 assay

The seeding of KGN cells was adopted in 96-well plate with 4000 cells/well. Determination of cell proliferation ability was via CCK-8 assay separately at 0, 24, 48 h on the grounds of the manufacturer’s instructions.

### 2.7. Plate cloning

The seeding of KGN cells was in a 6-well plate at a density of 500 cells/well. After about two weeks, cell colonies could be visible and then scrubbed twice via phosphate buffered saline (Sigma, St. Louis, Mo., USA). After the fixing via methanol (Sigma) and the staining via crystal violet (Sigma), count of colonies was under a microscope (Thermo Fisher Scientific).

### 2.8. The detection of cell apoptosis via flow cytometry

After the transfection and collection, the resuspension in buffer solution at a concentration of 1 × 10^6^/mL and staining with Annexin V-Fluorescein isothiocyanate and propidium iodide, the analysis of cell apoptosis was in FACSCalibur BD flow cytometer (BD Bioscience, USA).

### 2.9. The luciferase activity assay

The prediction and analysis of miR-29 c-3p with Foxo3ʹs targeting binding sites were via Bioinformatics website RNA22, and the cloning of corresponding sequences was into luciferase reporter vector pmirGLO (Promega Corp., Madison, WI, USA). The co-transfection of Foxo3-Wide type (WT)/mutant (MUT), miR-29 c-3p mimic/NC was into KGN cells. And then the evaluation of the relative luciferase activity was via a dual luciferase detection kit (Promega Corp., Madison, WI, USA).

### 2.10. RNA immunoprecipitation (RIP) assay

Magna RNA-binding protein immunoprecipitation kits (EMD Millipore, Billerica, MA, USA) were applied for determination of RIP. In brief, KGN cells were lysed in RIP buffer and then cell extract (100 mL) was co-incubated with magnetic beads coated with Anti-Argonaute2 (Anti-Ago2; Abcam, Cambridge, MA, USA) or immunoglobulin G (Anti-IgG; Abcam) at 4°C for 8 h. The samples were then detached with protease K (Solarbio) for 30 min. Finally, RNA in the complex was isolated and then RT-qPCR was performed to assess the enrichment of miR-29 c-3p and Foxo3.

### 2.11. The establishment and grouping of animal models

The selection of 10,021-day-old female Sprague Dawley rats (Shanghai Lab. Animal Research Center, China) was adopted as PCOS model, and other selection of 20 rats was as normal control group (the Sham). All rats were fed adaptively for 1 week in the experimental environment. The subcutaneous injection of PCOS model rats was with dehydroepiandrosterone (DHEA) (6 mg/100 g, dissolved in 0.2 mL soybean oil) for 21 d, while that of normal rats was with 0.2 mL soybean oil. From the 16th d, keratinocytes appeared continuously after the continuous 4-d observation of vaginal smear, which testified successful establishment of model. Next, the division of 120 model rats was into 6 random groups with each 20 rats. In these groups, caudal veins were injected with corresponding solution or plasmids. There was the model (injection of normal saline), the agomir NC (injection of agomir NC, 2.5 mg/kg/day), the miR-29 c-3p agomir (injection of miR-29 c-3p agomir, 2.5 mg/kg/day), the miR-29 c-3p agomir + pcDNA-NC (injection of miR-29 c-3p agomir + pcDNA-NC), the miR-29 c-3p agomir + pcDNA-Foxo3 (injection of miR-29 c-3p agomir + pcDNA-Foxo3). The rats of the normal group were injected with normal saline. Continuous administration of the drugs for 28 days was utilized. Gain of miR-29 c-3p agomir, agomir NC, pcDNA-Foxo3, and pcDNA-NC was from GeneBio (Shanghai, China) [23].

### 2.12. The detection of serum sex hormones and associated indexes of glucose metabolism

Ten randomly selected rats in each group were implemented with the anesthetization after the last injection. The chemiluminescence detection (Bayer, Leverkusen, Germany) of 5-mL blood taken from postcava was carried out to evaluate serum stimulating hormone (FSH), luteotropic hormone (LH), estradiol 2 (E2), prolactin (PRL), testosterone (T), and gonadotropin-releasing hormone (GnRH).

Ten randomly selected rats in each group were for glucose tolerance test. After the last injection and fast for 12 h, the extraction of orbital venous blood was conducted twice, one was after anesthesia with 3% pentobarbital sodium, the other was after intraperitoneal injection with 50% hypertonic glucose solution for 120 min. The measurement of blood glucose (BG), insulin (INS), insulin autoantibody (IAA), islet cell antibody (ICA) and glutamic decarboxylase antibody (GAD) was via automatic biochemical analyzer (Hitachi, Tokyo, Japan) and chemiluminescence detection system (Bayer, Leverkusen, Germany). The formula of resistance index of HOMA-IR is (fasting INS ˣ fasting blood glucose)/22.5.

### 2.13. Hematoxylin-eosin (HE) staining

After testing the indexes, six rats in each group were utilized with euthanasia. After removing the ovaries, the ovaries were adopted with the fixing with 4% paraformaldehyde the soaking in xylene, and then the absorption of xylene with distilled water. After embedding via paraffin, sectioning into thickness of 5 μm, dewaxing and the staining of the sections was adopted via hematoxylin and eosin. The dehydration was via alcohol and the elimination was via xylene, then the fixing was via neutral resin, and finally the observation was under a microscope (Olympus, Tokyo, Japan).

### 2.14. Statistical Methods

The processing of all the data was via SPSS 21.0 statistical software (SPSS, Inc, Chicago, IL, USA). And the measurement data were manifested as mean ± standard deviation (SD). The comparison between the two groups of measurement data subject to normal distribution was via t-test, and that of among multiple groups should be via one-way analysis of variance (ANOVA) and Tukey’s post hoc test. The comparison of data of different groups at different time points was via repetitive measured ANOVA and Bonferroni post hoc test. Pearson correlation was adopted to analyze miR-29 c-3p and Foxo3 in clinical samples. The indication of *P* < 0.05 was that the distinction has noteworthy statistical meaning.

## 3. Results

### 3.1. MiR-29 c-3p expression was down-regulated in peripheral blood of PCOS patients, while Foxo3 expression was up-regulated

**Figure 1.**
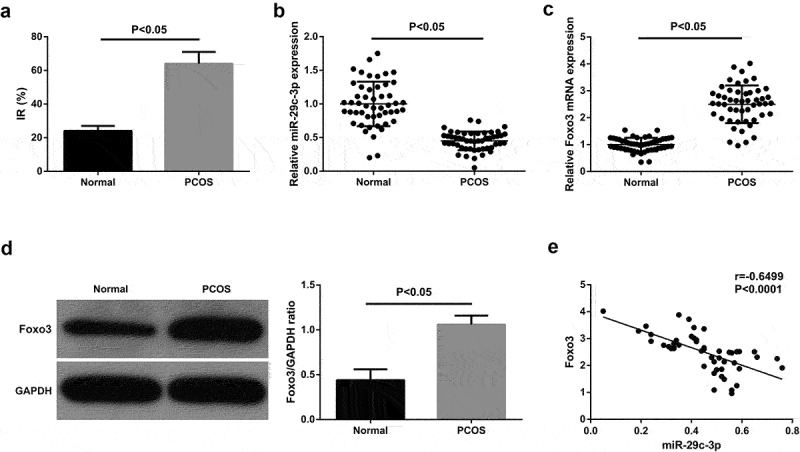
MiR-29 c-3p emerges declination in peripheral blood of PCOS patients, while Foxo3 presents oppositely (a) Incidence of IR in PCOS patients; B/C. The detection of miR-29 c-3p and Foxo3 via RT-qPCR; (d). The detection of Foxo3 via Western blot; E. The pertinence of miR-29 c-3p with Foxo3. The data in the Fig. were all measurement data, whose manifestation was as mean ± SD, *P* < 0.05.

Based on observations of metabolic spectrum of the subjects, it was discovered that the incidence of IR was ascending in PCOS patients (Figure 1(a)). Table 2 manifests the features of the subjects. It came out that miR-29 c-3p was silenced and Foxo3 was augmented in peripheral blood of PCOS patients (Figure 1(b-d)). And miR-29 c-3p was negatively associated with Foxo3 in peripheral blood of PCOS patients (Figure 1(e)).

**Table 2.** Clinical features of the PCOS and the control

Parameters	Normal (n = 50)	PCOS (n = 50)	P
Age (years)	30.60 ± 3.50	30.00 ± 3.60	0.22
BMI	21.22 ± 3.40	21.60 ± 3.20	0.45
Fasting Glucose (mmol/L)	5.0 ± 0.40	5.10 ± 0.50	0.28
Fasting Insulin (mU/L)	10.10 ± 3.60	14.20 ± 5.20	<0.05
HOMA-IR	2.10 ± 0.80	3.30 ± 2.40	<0.05
T (nmol/L)	1.20 ± 0.30	1.50 ± 0.50	<0.05
FSH (IU/L)	7.00 ± 1.80	6.60 ± 1.40	<0.05
LH (IU/L)	4.70 ± 1.60	7.80 ± 5.20	<0.05

### 3.2. Elevated miR-29 c-3p facilitates granulosa cell advancement, while the silenced one emerges inverse effect

**Figure 2.**
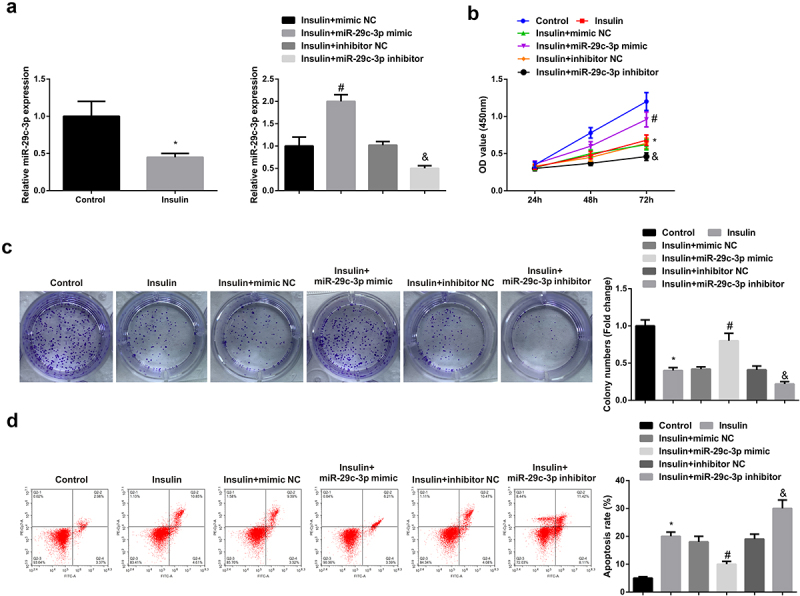
Elevated miR-29 c-3p motivates granulosa cell advancement, while the silenced one emerges inverse effect.(a). The detection of miR-29 c-3p in KGN cells via RT-qPCR; (b-c). The detection of cell proliferation via CCK-8 and plate cloning; D. The detection of cell apoptosis via flow cytometry. The data in the Fig. were all measurement data, whose manifestation was as mean ± SD (n = 3), * vs the control, *P* < 0.05; # vs the mimic NC, *P* < 0.05; & vs the inhibitor NC, *P* < 0.05.

The transfection of mimic/inhibitor NC and miR-29 c-3p mimic/inhibitor was into KGN cells for the sake of exploring the impacts of miR-29 c-3p on the biological function of KGN cells, with the verification of the successful transfection (Figure 2(a)). Cell proliferation was detected by CCK-8 and colony formation assay, and it was found that up-regulation of miR-29 c-3p could promote KGN cell proliferation (Figure 2(b,c)). Cell apoptosis was detected by flow cytometry, and experimental results showed that up-regulation of miR-29 c-3p could inhibit apoptosis of KGN cells (Figure 2(d)). However, after down-regulation of miR-29 c-3p, its biological function was restrained (Figure 2(b-d)). The data illustrated that elevated miR-29 c-3p accelerated granulosa cell advancement, while the silenced one emerged the inverse effect.

### 3.3. MiR-29 c-3p negatively modulates Foxo3

**Figure 3.**
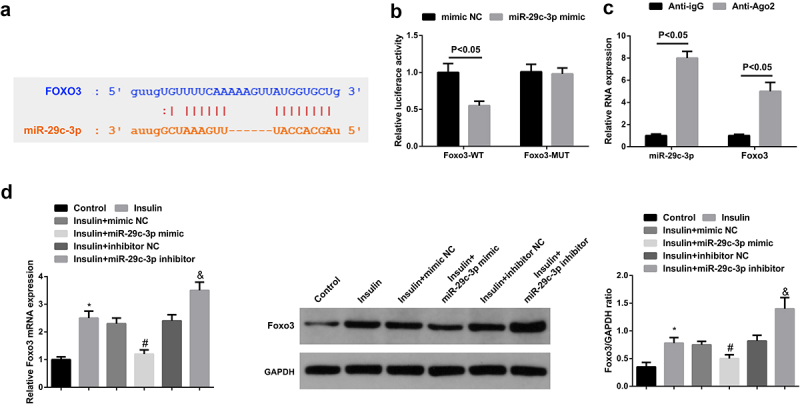
MiR-29 c-3p negatively mediates Foxo3. (a). The targeting sites of miR-29 c-3p and Foxo3; (b). The validation of targeting connection of miR-29 c-3p with Foxo3 via the luciferase activity assay; (c): RIP assay detection of the enrichment of miR-29 c-3p and Foxo3 in Anti-Ago2 or Anti-IgG immunoprecipitation complex in KGN cells; (d). The detection of Foxo3 via RT-qPCR and Western blot. The data in the Fig. were all measurement data, whose manifestation was as mean ± SD (n = 3).

It was known that silenced miR-29 c-3p and augmented Foxo3 were presented in the peripheral blood of PCOS patients with IR, and the two were negatively associated, thus a targeting connection of miR-29 c-3p with Foxo3 was suspected. After the prediction of the binding site of the targeting of miR-29 c-3p with Foxo3 via bioinformatics website (Figure 3(a)), the result of the luciferase activity assay delineated the prominent declination of the relative luciferase activity after the co-transfection of Foxo3-WT with miR-29 c-3pmic (Figure 3(b)), and RIP assay results showed that the levels of miR-29 c-3p and Foxo3 were significantly increased in the Anti-AgO2 immunoprecipitation complex in KGN cells (Figure 3(c)). Next, it was observed in the detection of Foxo3 in KGN cells that augmented miR-29 c-3p triggered decreasing Foxo3 while silenced one incurred ascending Foxo3 (Figure 3(d)). In summary, miR-29 c-3p repressed Foxo3 expression in KGN cells via direct targeting.

### 3.4. Augmented Foxo3 reverses the function of miR-29 c-3p

**Figure 4.**
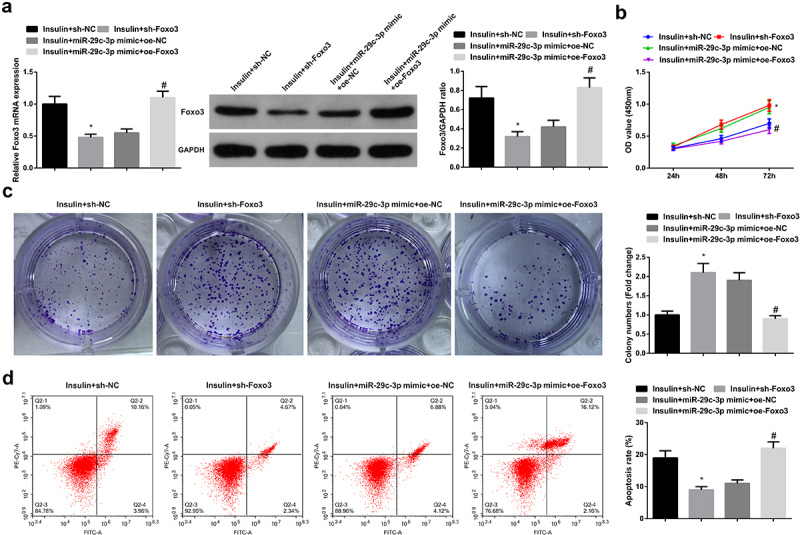
Elevated Foxo3 reverses the function of miR-29 c-3p. (a). The detection of Foxo3 via RT-qPCR and Western blot; B-C. The detection of cell proliferation via CCK-8 and plate cloning; D. The detection of cell apoptosis via flow cytometry. The data in the Fig. were all measurement data, whose manifestation was as mean ± SD (n = 3), * vs the sh-NC, *P* < 0.05; # vs the miR-29 c-3p mimic + oe-NC, *P* < 0.05.

The transfection of sh-NC/Foxo3, miR-29 c-3p mimic + oe-NC/Foxo3 was into KGN cells for the sake of exploring the impacts of Foxo3 on the biological function of KGN cells. The results showed that downregulation of Foxo3 could promote KGN cell proliferation but inhibit apoptosis. After up-regulation of Foxo3, KGN cell proliferation ability was inhibited and apoptosis was increased (Figure 4(a-d)).

### 3.5. Augmented miR-29 c-3p ameliorates the production of serum sex hormones and glucose metabolism in PCOS rats, while elevated Foxo3 reverses the improvement impact of miR-29 c-3p

**Figure 5.**
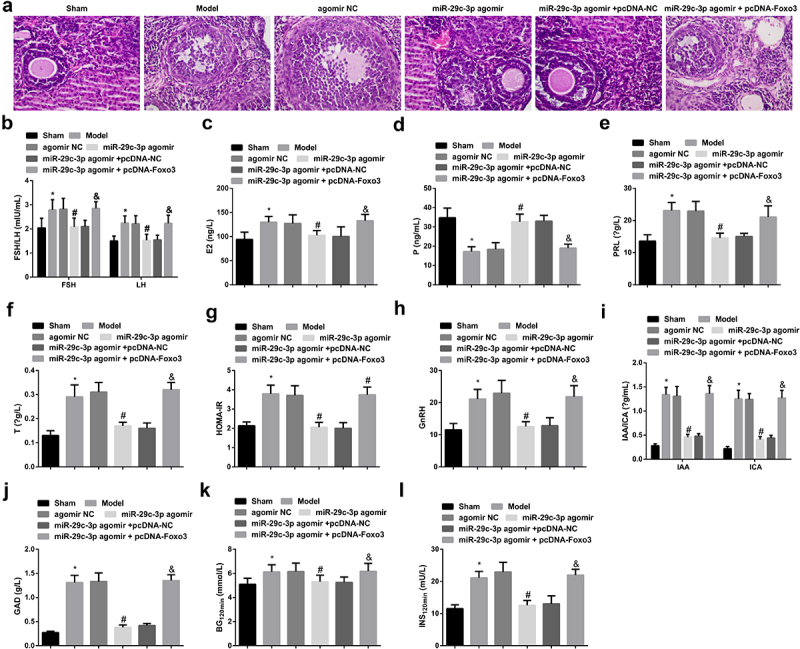
Augmented miR-29 c-3p ameliorates the production of serum sex hormones and glucose metabolism in PCOS rats, while elevated Foxo3 turns around the improvement of miR-29 c-3p. (a). Detection of PCOS development via HE staining; B-L. The production of serum sex hormones and glucose metabolism in PCOS rats. The data in the Fig. were all measurement data, whose manifestation was as mean ± SD (n = 5), * vs the Sham, *P* < 0.05; # vs the agomir NC, *P* < 0.05; + vs the miR-29 c-3p agomir + pcDNA-NC, *P* < 0.05.

For the sake of further verifying the function of miR-29 c-3p on PCOS *in vivo*, the establishment of PCOS rat model was put into use. It was observed that ovarian tissue, oocytes and granulosa cells in the Sham were all normal. The model manifested miscellaneous subcapsular follicular cysts with very thin or no granular cells and the disappearance of oocytes (Figure 5(a)), which elucidated that PCOS rat model was a success and could be applied for subsequent experiments.

Then, the study of underlying influences of miR-29 c-3p was utilized on serum sex hormone production and glucose metabolism in PCOS rats. HOMA-IR in the Sham was below 2.69, indicating that there was no IR in it. In the Model, FSH, LH, E2, P, PRL, T, HOMA-IR, BG_120min_, INS_120min_, GnRH, IAA, ICA, and GAD were augmented, but P was decreased. After the elevation of miR-29 c-3p, P escalated while other biochemical indexes reduced. And augmented Foxo3 reversed the function of miR-29 c-3p (Figure 5(b-l)). To sum up, augmented miR-29 c-3p ameliorated the production of serum sex hormones and glucose metabolism in PCOS rats, while elevated Foxo3 reversed the improvement of miR-29 c-3p.

## 4. Discussion

PCOS is now considered as a momentous metabolic and reproductive disease, and women with PCOS tend to suffer long-term health problems [24]. Due to the increasing incidence of PCOS, PCOS has been widely concerned by researchers. Studies have shown that insulin is inclined to be rather crucial in motivating or maintaining PCOS [25,26]. IR/hyperinsulinemia is a considerable external element in numerous PCOS cases, giving rise to pseudoacromegaly, lipogenesis, steroidogenesis, hyperandrogenism, and lipid metabolism disorder [27–29]. Meanwhile, the occurrence of PCOS is related to the growth, survival, and apoptosis of ovarian granulosa cells [30,31]. KGN cells have most of the physiological characteristics of ovarian cells. In this study, the effects of IR/hyperinsulinemia on KGN cell proliferation and apoptosis were investigated. It was found that high concentrations of insulin inhibited granulosa cell proliferation and induced apoptosis.

Hyperinsulinemia is linked with the pathogenesis of PCOS [32]. Studies have elucidated that hyperinsulinemia or excessive secretion of Luteinizing Hormone (LH) is able to induce abnormal response of granulosa cells to LH and thus influencing the development of follicle [33–35]. The therapy of PCOS with insulin-reducing drugs is able to mitigate ovarian function and lessen androgen [36]. Nevertheless, it has not been clarified that the mechanism of the contradiction of insulin sensitivity with IR coexistence in controlling granular cells. In this study, it was observed the depression of granulosa cell progression after the treatment with 0.5 mmol/L insulin. In addition, a study has suggested that patients with nonalcoholic steatohepatitis and chronic hepatitis C virus infection have impaired insulin signaling and increased cell apoptosis in the liver [37]. Dysfunctional insulin signal is able to restrain retinal Müller cell apoptosis [38]. These results further validated the perspective that insulin is implicated in modulating granulosa cell progression.

In the meantime, this study also uncovered that miR-29 c-3p expression was decreased but Foxo3 expression was increased in PCOS patients. And it was illustrated that miR-29 c-3p was negatively associated with Foxo3 in PCOS patients. Foxo3 was confirmed to be the direct target gene of miR-29 c-3p by bioinformatics website, dual-luciferase assay, and RIP assay. It is worth noting that the description that miRNAs participate in the development of diseases is introduced in previous studies. For instance, miR-29 c-3p manifests anti-inflammatory properties in PD animal and neuron models [39]. A previously reported study clarifies that Foxo3 is augmented in PCOS and represses the progression of ovarian granulosa cells [22], which is consistent with the results in the study, that is, augmented miR-29 c-3p or silenced Foxo3 promoted granulosa cell proliferation but inhibited cell apoptosis.

Interestingly, it was also observed that the elevation of miR-29 c-3p improved the production of serum sex hormones and glucose metabolism in PCOS rats, for the grounds of giving rise to a declination in the production of FSH, LH, E2, PRL, T, HOMA-IR, BG_120min_, INS_120min_, GnRH, IAA, ICA and GAD, but an augmentation in the production of P. But augmented Foxo3 was able to reverse the impacts of elevation of miR-29 c-3p on serum sex hormone production and glucose metabolism in PCOS rats.

And yet there are still some limitations in this study. First, further study is required to determine whether Foxo3 has a more specific function under the impact of insulin and to learn about its mechanism. Besides, apart from IR/hyperinsulinemia in the collected cases, there are other elements that cannot be correctly evaluated in this study. As a consequence, more appropriate cases are badly in need of later for the sake of exploring the function of IR/hyperinsulinemia more accurately.

## 5. Conclusion

This study elucidates that miR-29 c-3p is able to participate in insulin function via targeting Foxo3, facilitate granulosa cell advancement *in vitro*, recover the sex hormone of PCOS rats and ameliorate glucose metabolism *in vivo*. All in all, this study manifests the mechanism that miR-29 c-3p is implicated in insulin function in PCOS mediation via targeting Foxo3.
